# Integrated Analysis of the Transcriptome and Metabolome of *Cecropia obtusifolia*: A Plant with High Chlorogenic Acid Content Traditionally Used to Treat Diabetes Mellitus

**DOI:** 10.3390/ijms21207572

**Published:** 2020-10-14

**Authors:** Jorge David Cadena-Zamudio, Pilar Nicasio-Torres, Juan Luis Monribot-Villanueva, José Antonio Guerrero-Analco, Enrique Ibarra-Laclette

**Affiliations:** 1 Instituto de Ecología, A.C. (INECOL), Red de Estudios Moleculares Avanzados (REMAV), Xalapa 91073, Veracruz, Mexico; cadenazamudioj@gmail.com (J.D.C.-Z.); juan.monribot@inecol.mx (J.L.M.-V.); joseantonio.guerrero@inecol.mx (J.A.G.-A.); 2 Instituto Mexicano del Seguro Social (IMSS), Centro de Investigación Biomédica del Sur (CIBIS), Xochitepec 62790, Morelos, Mexico; pisaliva@yahoo.com.mx

**Keywords:** *Cecropia obtusifolia* (guarumbo), chlorogenic acid, plant metabolomics, RNA-seq, diabetes mellitus

## Abstract

This investigation cultured *Cecropia obtusifolia* cells in suspension to evaluate the effect of nitrate deficiency on the growth and production of chlorogenic acid (CGA), a secondary metabolite with hypoglycemic and hypolipidemic activity that acts directly on type 2 diabetes mellitus. Using cell cultures in suspension, a kinetics time course was established with six time points and four total nitrate concentrations. The metabolites of interest were quantified by high-performance liquid chromatography (HPLC), and the metabolome was analyzed using directed and nondirected approaches. Finally, using RNA-seq methodology, the first transcript collection for *C. obtusifolia* was generated. HPLC analysis detected CGA at all sampling points, while metabolomic analysis confirmed the identity of CGA and of precursors involved in its biosynthesis. Transcriptome analysis identified differentially expressed genes and enzymes involved in the biosynthetic pathway of CGA. *C. obtusifolia* probably expresses a key enzyme with bifunctional activity, the hydroxycinnamoyl-CoA quinate hydroxycinnamoyl transferase and hydroxycinnamoyl-CoA shikimate/quinate hydroxycinnamoyl transferase (HQT/HCT), which recognizes shikimic acid or quinic acid as a substrate and incorporates either into one of the two routes responsible for CGA biosynthesis.

## 1. Introduction

Plant secondary metabolites (SMe) are compounds with a restricted presence in taxonomic groups that participate in the interaction of cells with the environment to ensure the survival of the organism and provide an evolutionary advantage in its ecosystem [1,2]. SMe exhibit great structural variation, and their production is often associated with biotic or abiotic stress. Consequently, a wide range of biological activities are exhibited, such as antimicrobial, antigerminative, toxic, and attractant properties or symbiont interaction. These features have allowed researchers to identify more than 200,000 plant SMe, largely represented by terpenes, alkaloids, and polyphenols [3,4]. Polyphenols have received considerable attention due to their pharmacological and biomedical potential in chronic human degenerative diseases such as cataracts, macular degeneration, endothelial dysfunction, arthritis, cancer, and diabetes mellitus [5]. Many recent studies have focused on finding new phytomedicines with greater specificity, greater human safety and sometimes a lower economic cost to provide new options for both the pharmaceutical industry and patients [6,7]. We highlight *Cecropia obtusifolia* Bertol (Urticaceae), an arboreal species popularly known as guarumbo in Mexico and traditionally used in the medical treatment of diabetes mellitus type 2 (DM-2). Infusions of leaves, branches, bark, and roots are used for their hypoglycemic and hypolipidemic properties demonstrated in experimental animal and human models, both at a cellular and physiological level, which are attributed to chlorogenic acid (CGA), making *C. obtusifolia* a model plant for its potential use in the control of DM-2 [8,9,10,11,12,13,14]. In the same way, it is known that *C. obtusifolia* is capable of synthesizing, in addition to CGA, other compounds of phenolic origin such as orientine, isoorientine, vitexin, isovitexin, caffeic acid, ferulic acid, quinic acid, quercetin, apigenin, luteolin, tormentoside, triterpenoid isomers of saponin-O-hexoside and chlorogenic acid, among others, which are known to be present in the leaves of the plant [15,16,17]. Studies on *C. obtusifolia* cell cultures have shown that nitrate starvation increases CGA production [18]. Despite the importance of this plant, no omics studies were previously available. Therefore, the objective of this research was to evaluate the effect of nitrate restriction in *C. obtusifolia* cell suspension cultures to identify genes and proteins involved in the biosynthesis of CGA. This transcriptomic/metabolomic study represents the first collection of transcripts reported for this species. According to the chemical-analytical and computational-theoretical analyses performed, the *C. obtusifolia* CGA biosynthesis pathway is proposed.

## 2. Results

### 2.1. Effect of Nitrate Starvation on the Proliferation Kinetics of C. obtusifolia Cells

**Figure 1:**
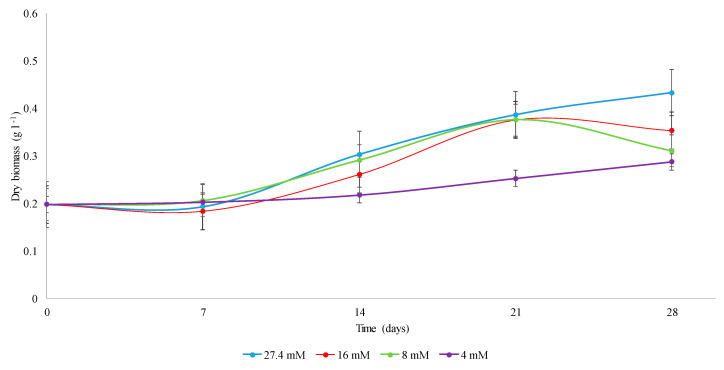
Kinetics of proliferation of cell suspensions of *C. obtusifolia* grown in Murashige and Skoog (MS) medium supplemented with different concentrations of nitrates (27.4, 16, 8 and 4 mM). The dispersion bars represent the standard deviation calculated from three biological replicates that were used for each sampling point analyzed over the time course.

The *C. obtusifolia* cell suspension cultures at 16 and 8 mM nitrates in Murashige and Skoog (MS) medium showed a similar proliferation rate to the control during the exponential phase (7–21 days). However, at later evaluation times (28 days), both stress conditions provoked a decay, which suggests a reduction in the rate of cell proliferation and viability after prolonged stress (Figure 1). Under the most restricted nitrate condition (4 mM), cell proliferation was severely affected (Figure 1). 

No differences in cell proliferation were observed under any stress condition at early stages (zero and three hours), as confirmed by Student’s *t* analysis for independent samples (*p* value = 0.2058). This was expected due to the rapid doubling rate generally observed for plant cells in suspension cultures and mainly because these times represent the adaptation phase. Although a clear reduction in the average maximal biomass obtained was observed at 4 mM (Table 1), there were no statistically significant differences in the kinetic constants (F_0.05_ = 2.82, *p* = 0.1075). However, as expected, Tukey’s test indicated that the differences in the duplication time (F_0.05_ = 21.01, *p* = 0.0004) and in the specific cell growth rate μ (F_0.05_ = 11.05, *p* = 0.0032) at 4 mM were significant with respect to the other concentrations (27.4, 16 and 8 mM). 

### 2.2. Determination of Phenolic Compounds by Targeted Metabolomics

CGA in the *C. obtusifolia* cell cultures was identified and quantified by HPLC analysis. Retention times of 13.299 for commercial standard and 13.454 for CGA in cell cultures, which are shown in Supplementary Figure S1a,b, were determined. The results show that CGA is detectable throughout the entire kinetics time course and in all nitrate concentrations evaluated, see Supplementary Figure S2; moreover, the CGA identity was corroborated by enrichment analysis of one of the samples using the standard to perform a coelution, see Supplementary Figure S3a,b. Unlike other studies where nitrate deficiency stress induces isorientin accumulation, in this study, it was not possible to identify this SMe under any of the conditions tested.

In addition, targeted metabolomic analysis was performed, and phenolic compounds were identified and quantified based on the reference mass spectra and retention times of 60 commercial standards, where the main phenolic compounds identified in the different nitrate treatments (27.4, 16 and 4 mM) and their dynamics over time (three hours, 7, 14, 21, and 28 days) stood out (Table 2), as shown in Supplementary Figure S4a–n. In Supplementary Table S1, a list of all phenolic compounds identified and quantified is shown. We also identified chemical compounds involved in CGA biosynthesis in the phenylpropanoid pathway. CGA was the major phenolic compound determined in *C. obtusifolia* cell suspensions. A CGA accumulation tendency was observed along with cell proliferation in nitrate deficiency treatments (16 and 4 mM), reaching the highest levels at 21 and 28 days. Under normal nitrate conditions, a slight tendency to decrease over time was observed (except at 21 days). Shikimic, *t*-cinnamic, *p*-coumaric and caffeic acids are chemical compounds involved in upstream of CGA biosynthesis. Shikimic and *t*-cinnamic acids were not detected at any sampling time under normal nitrate conditions, while in the nitrate deficiency treatments (16 and 4 mM), shikimic acid was detected at 7 and 14 days, respectively. *t*-Cinnamic acid was detected only under 16 mM nitrate at 21 and 28 days, and *p*-coumaric acid was detected at the first two time points (three hours and seven days) under 4 mM nitrate and at 28 days under normal conditions. Caffeic acid was detected under all nitrate conditions at all sampling times and shared the same tendencies as CGA in all time course experiments. Under the control nitrate condition, a slight decrease was observed, while the opposite occurred under nitrate deficiency conditions (16 and 4 mM). The difference in caffeic acid concentration at 16 mM nitrate was significantly higher than at 27.4 mM nitrate starting at 21 days. Ferulic acid, vanillin, vanillic acid and quercetin-3-D-galactoside are phenolic compounds downstream of CGA biosynthesis. Ferulic acid was detected at three hours under 4 mM nitrate deficiency and at seven days at 27.4 and 16 mM nitrate. At 27.4 and 16 mM, a slight increase was observed over time, while at 4 mM nitrate, an increase followed by a decrease was observed after 14 days. In all nitrate conditions, vanillin showed a slight decrease over time, reaching its minimum at 14 days and then an increase after 21 days. Vanillic acid was detected in all nitrate treatments at several sampling times without a clear tendency. Finally, quercetin-3-d-galactoside was detected in normal and nitrate-deficient treatments at seven days and three hours, respectively. In all nitrate treatments, an upward trend was noticeable through the time course experiment.

### 2.3. Identification of Chemical Markers by Untargeted Metabolomics Analysis

**Figure 2:**
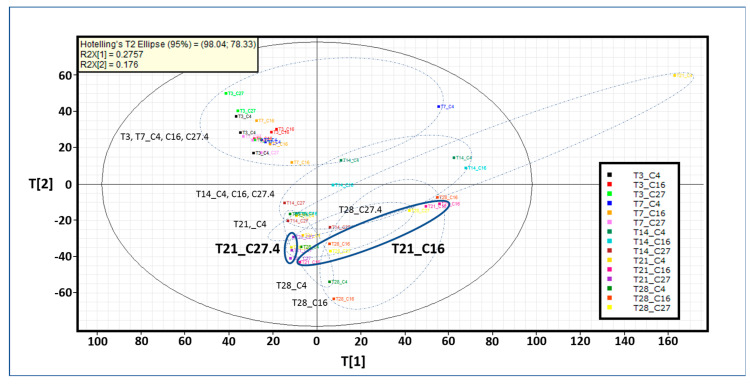
Untargeted metabolomics analysis. Principal component analysis (PCA) mediated grouping based on the chemical profiles of suspension cell cultures of *C. obtusifolia* cultured in normal nitrate (27.4 mM) and nitrate-deficient (16 and 4 mM) conditions over the sampling times (3 h, 7, 14, 21 and 28 days). Each point in the plot corresponds to an observation (sampling time_nitrate concentration). “T” and “C” represent time and concentration, respectively. T3, T7, T14, T21 and T28 represent the sampling times of 3 h, 7, 14, 21 and 28 days, respectively. C27, C16 and C4 represent nitrate concentrations of 27.4, 16 and 4 mM, respectively. The chemical profile dataset consisted of 2807 exact mass/retention time (EMRTs). T[1], first principle component score and T[2], second principle component score (x- and y-axis respectively), are the two most important new variables that summarize the dataset and together explain 45.17% of the dataset variance. The different groups are surrounded by dashed lines. The T21_C27.4 and T21_C16 samples are outlined to highlight the treatments used for further statistical analysis.

**Figure 3:**
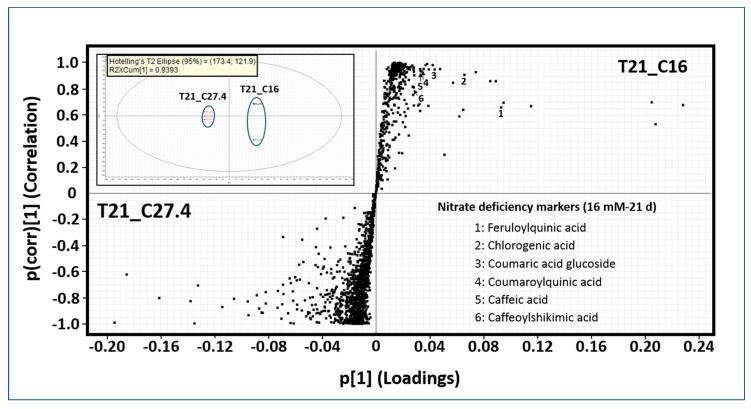
S-plot comparing the chemical profiles of T21_C27.4 and T21_C16 samples. The upper right quadrant shows the sample grouping after orthogonal partial least squares discriminant analysis (OPLS-DA). The upper right quadrant of the S-plot shows the components that are elevated in T21_C16, while the lower left quadrant shows the exact mass/retention time (EMRTs) elevated in T21_C27.4. The distance along the x-axis corresponds to the contribution to the variance between the groups, while the distance along the y-axis corresponds to the reliability of the analytical result. The lower right quadrant of the S-plot shows some T21_C16 chemical markers tentatively identified as phenolic compounds based on accurate mass spectrometry.

The targeted metabolomics analysis was performed with 60 standards, most of them involved upstream and downstream of CGA biosynthesis in the phenylpropanoid pathway. We were capable of accurately quantifying nine of the main precursors of CGA (Table 2). Therefore, to address this question, untargeted metabolomics analysis was performed. Principal component analysis (PCA) shows the groupings for all nitrate conditions over time constructed with 2807 exact mass/retention time pairs (EMRTs) (Figure 2). T[1] (first principle component score) and T[2] (second principle component score) are the two most important variables that summarize the dataset and together explain 45.17% of the dataset variance. Chemical profiles under any nitrate conditions are very similar at early time points (three hours and seven days). At 14 days, the chemical profiles differ from those observed at three hours and seven days but are similar under all nitrate conditions. Major differences were observed at later times, mainly at 21 days. Therefore, further statistical analysis was performed at 21 days comparing normal (27.4 mM) and nitrate-deficient (16 mM) conditions (Figure 3). The upper left quadrant shows the grouping after the statistical analysis performed, and both nitrate treatment samples are grouped separately. Each point in the upper right and lower left quadrants of the orthogonal partial least squares discriminant analysis (OPLS-DA) S-plot represents an EMRT where the farther the position along the x- and y-axes is, the greater the contribution to the variance between the groups and the higher the reliability of the analytical result are, respectively. These EMRTs are considered to represent chemical markers. In the nitrate deficiency treatment (right upper quadrant) at 21 days, some EMRTs were tentatively identified as phenolic compounds based on their exact mass spectrometric fingerprints (lower right quadrant), see Supplementary Table S2. Caffeic acid and CGA were previously identified and quantified by the targeted metabolomics approach, and the compound concentrations were higher under the nitrate-deficient condition (16 and 4 mM) than under the normal nitrate treatment (27.4 mM). Feruloylquinic acid and coumaric acid glucoside are phenolic compounds apparently not involved in CGA biosynthesis, while coumaroylquinic and caffeoylshikimic acids are direct precursors of CGA.

### 2.4. Construction of the Unigene Set Generated from RNA Isolated from C. obtusifolia Cell Suspensions

A total of 556,632,315 paired-end reads were generated from the 45 libraries that were sequenced. On average, 37,108,821 paired-end reads were generated for each of the libraries, one for each concentration of nitrates at each time (three hours, 7, 14, 21, and 28 days) in all the time courses tested. Each of the three sequenced biological replicates contributed approximately 12,369,607 million paired reads. Before *de novo* assembly, the raw reads were filtered to include only those with acceptable quality according to the established threshold. In addition, “long sequences” were generated by merging the paired-end reads that showed overlapping end regions, see Supplementary Table S3. Both sets of data (long sequences and nonmerged paired-end reads) were used to assemble the transcriptome of *C. obtusifolia* (see methods for more details). In total, 222,691 unigenes were generated, ranging between 200 and 17,269 bp with an average length of 245 bp.

The out-of-frame insertions/deletions in the coding regions were corrected using the AlignWise pipeline [19] (Supplementary Materials for more details). After the frameshift was corrected, the redundant sequences were eliminated. A sequence was considered redundant if it presented at least 95% identity with respect to another unigene, maintained along 90% or more of the sequence length. The corrected and nonredundant unigene set comprised curated transcripts from *C. obtusifolia* with a total of 118,756 unigenes, whose coding regions can be translated into proteins/peptides, shown in Supplementary Table S4. Only these sequences were considered for future analysis.

### 2.5. Annotation and Functional Categorization of the C. obtusifolia Transcriptome

**Figure 4:**
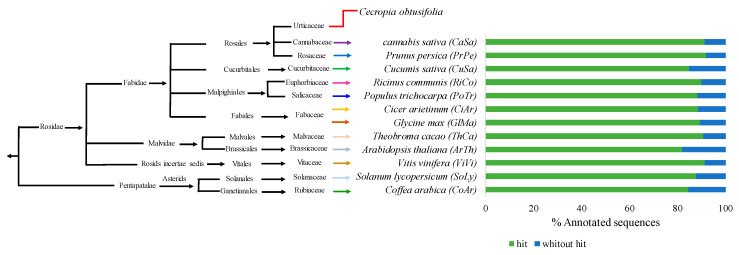
Percentage of *C. obtusifolia* unigenes annotated by identification of protein homologs from other plant species. BLASTp similarity searches were performed using the translated coding sequence (CDS) identified in *C. obtusifolia* nonredundant unigenes. Reference proteomes correspond to some angiosperm plant species selected based on previous reports that confirm their chlorogenic acid (CGA) content. These species are shown according to their relationship and evolutionary history (*Cannabis sativa* (CaSa); *Prunus persica* (PrPe); *Cucumis sativus* (CuSa); *Ricinus communis* (RiCo); *Populus trichocarpa* (PoTr); *Cicer arietinum* (CiAr); *Glycine max* (GlMa); *Theobroma cacao* (ThCa); *Arabidopsis thaliana* (ArTh); *Vitis vinifera* (ViVi); *Solanum lycopersicum* (SoLy); *Coffea arabica* (CoAr)).

Most of the proteins translated from the coding regions identified in the nonredundant unigenes of *C. obtusifolia* (a total of 111,306 proteins) showed significant similarity (value- e < 10^−5^) with proteins of at least one of the twelve plant species against which they were compared. The percentage of annotated unigenes according to the species compared ranged between 81.94% and 92.01% (Figure 4). With the exception of two species used as references (*Vitis vinifera* and *Theobroma cacao*), the percentage of *C. obtusifolia* unigenes that can be annotated as a function of other species varies with the phylogenetic relationship (Figure 4).

Regarding the functional categorization of the *C. obtusifolia* unigenes, Gene Ontology (GO) terms were assigned based on the information on the homologs identified in the *Arabidopsis thaliana* genome, see Supplementary Table S5. A total of 5607 unigenes (2.51% of the total) were assigned to at least one or more distinct subcategories or GO terms. All of them belonged to some of the three major categories (“biological process”, represented by 392 GO terms; “molecular function”, 107 GO terms; and “cellular components”, 82 GO terms).

### 2.6. Identification of C. obtusifolia Genes Differentially Expressed in Response to Nitrate Starvation

**Figure 5:**
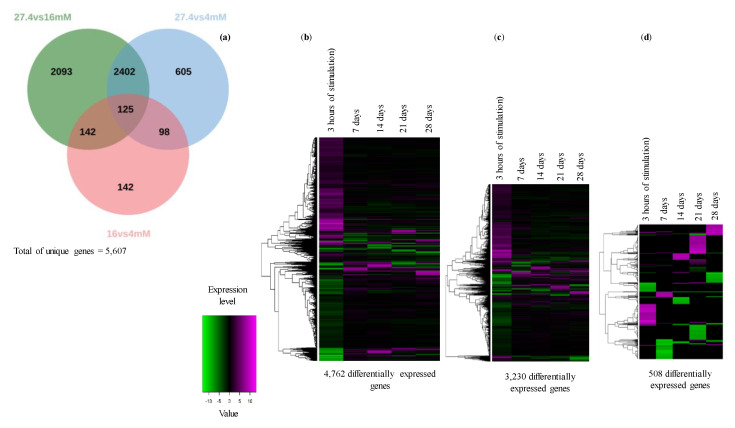
Venn diagram (**a**) shows the numbers of common and specific differentially expressed genes (DEGs) identified over time in pairwise sample comparisons, for example, the control treatment (27.4 mM) versus nitrate deficiency (16 mM). The heat maps represent a hierarchical clustering analysis of DEGs identified after pairwise comparison of different treatments: 27.4 mM vs 16 mM (**b**); 27.4 mM vs 4 mM (**c**); 16 mM and 4 mM (**d**). The heat maps in the figure were constructed using heatmap.2 in the “gplots” package of R [22]. Log_2_-transformed fold change values were calculated for the indicated sampling points (3 h, 7, 14, 21 and 28 days). Horizontal rows represent individual unigenes, and vertical columns represent time points. As shown on the color scale at the bottom of the figure, blue indicates downregulated (−), red indicates upregulated (+), and white indicates unchanged values.

To compare the expression levels of the *C. obtusifolia* unigenes in each of the samples analyzed and to identify those that are differentially expressed in response to nitrate starvation, the RNA-seq by Expectation-Maximization pipeline (RSEM) [20] and DEseq software were used [21]. First, an expression profile matrix was created, which included 111,306 annotated unigenes (rows) and the number of expected reading counts calculated in each of the generated libraries (columns). To make the expected reading count values across the samples comparable, RSEM also calculates transcripts per million (TPM) and fragments per kilobase of contigs/unigenes per million mapped reads (FPKM) values, see Supplementary Table S6. In this study, the value of FPKM was chosen as the representative value of the expression profiles. The differentially expressed genes (DEGs) were identified once expected reading counts were normalized through a pairwise comparison using the sampling points (three hours, 7, 14, 21 and 28 days) at 27.4 mM as a control and comparing them against those at 16 mM and 4 mM, respectively. A total of 5606 unigenes were selected as differentially expressed in at least one of the comparisons made. A Venn diagram with the DEGs showed that the conditions 27.4 mM versus 16 mM had the highest number of DEGs (4762), followed by 27.4 mM vs 4 mM, with 3230 genes, 16 mM vs 4 mM with 508 genes (Figure 5a), shown in Supplementary Table S7. To obtain a graphic representation of how the DEGs respond to nitrate deficiency over time, heatmaps based on a hierarchical clustering approach were created (Figure 5b–d). Interestingly, when the 16 or 4 mM stress condition was compared with the control condition (27.4 mM), it was evident that most of the DEGs responded to stress positively or negatively at early time points (three hours), and the changes became less evident later (Figure 5b–d).

### 2.7. Gene Ontology Enrichment Analysis of the DEGs

**Figure 6:**
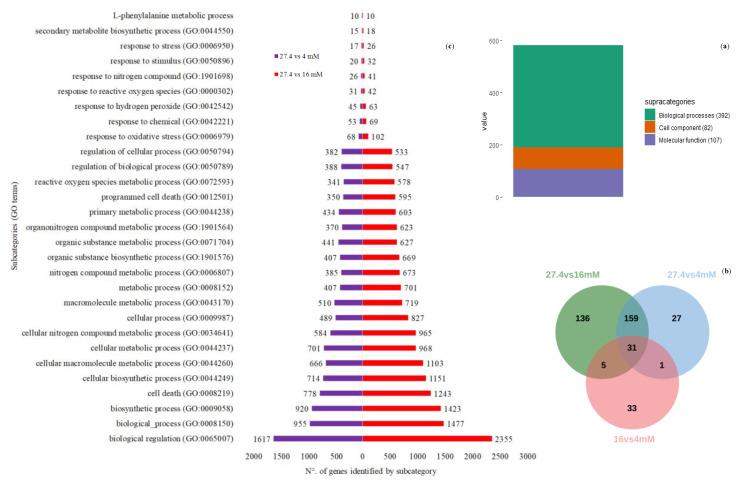
Pie chart (**a**) showing the distribution of the 587 subcategories identified within the 3 different Gene Ontology (GO) major categories. All these subcategories resulted from analysis of the enrichment of GO terms in the lists of differentially expressed genes. The Venn diagram (**b**) depicts the number of shared and unique functional subcategories belonging to the “biological process” category resulting from the differentially expressed genes identified by pairwise comparison of treatments (27.4 mM vs 16 mM, 27.4 mM vs 4 mM, 16 mM vs 4 mM). Finally, (**c**) shows some GO terms enriched with the highest number of grouped differentially expressed genes. Some of these categories are discussed and can explain (at least in part) the biological phenomenon of nitrate deficiency responses evaluated in this study.

To identify the functional categories enriched in the DEGs, the Gene Ontology (GO) tool [23] was used [24]. A total of 581 functional categories were enriched, including 392 (67.4%), 107 (18.4%) and 82 (14.1%) categories belonging to biological processes, molecular functions and cellular components, respectively (Figure 6a and Supplementary Table S5). The largest number of enriched GO terms was displayed from the list of DEGs identified in the 16 mM vs 27.4 mM contrast. However, it is important to note that a large number of GO terms are shared after comparing the enriched functional categories on the lists of DEGs, which were selected after comparing any of the nitrate deficiency treatments (16 and 4 mM) vs the control condition (27.4 mM). As expected, only a small number of enriched categorical results from the list of DEGs resulted from comparing the two conditions that represented the use of abiotic stress (nitrate starvation) (Figure 6b). Some subcategories resulted from our special interest (Figure 6**c**). For example, the subcategory “response to nitrate” (GO: 0010167) includes proteins such as NRT2.5 (AT1G12940) with a high-affinity nitrate transporter [25], and the categories “response to reactive oxygen species” (GO: 0000302) and “reactive oxygen species metabolic process” (GO: 0072593) show results consistent with previous reports showing that hydrogen peroxide modulates root cell responses to nutrient deprivation, including nitrogen deprivation [26]. Related to the last categories described, it is also important to emphasize the presence of some specific genes, e.g., the “response to oxidative stress” category. This group includes the unigene UN063823, a homolog of peroxirredoxin (PRX, AT3G52960), an enzyme involved in hydrogen peroxide detoxification [27]. In addition, the unigenes UN069212, UN075426, UN076799 and UN033315 encode peroxidase protein homologs (AT5G15180, AT2G39040 and AT5G05340) and ascorbate peroxidases (ATG52880) [28,29,30,31,32,33]. Subcategories such as “cell death” (GO: 0008219) and “programmed cell death” (GO: 0012501) attracted our attention due to their relationship with cell viability, which is compromised at the endpoints sampled at 8 and 16 mM. Another subcategory, the “L-phenylalanine metabolic process” (GO: 0006558), appears enriched in the comparison of 27.4 mM versus 16 mM, but not 27.4 mM versus 4 mM. This suggests that compounds that use L-phenylalanine as a biosynthetic intermediate can be better induced under moderate stress conditions. The unigenes UN059693, UN073057, UN061336 and UN064046, homologues of the enzyme phenylalanine ammonia lyase (PAL), are part of a multigenic family involved in an interesting defense network in plants, which is induced in response to various environmental stresses, such as nutrient deficiency [34,35,36]. The expression of some members of this family is correlated with the induction of genes involved in the defense response of plants and in the response to oxidative stress [35,37].

### 2.8. Identification of Orthologous Genes and Search for Unigenes of C. obtusifolia Encoding Enzymes Involved in CGA Synthesis

**Figure 7:**
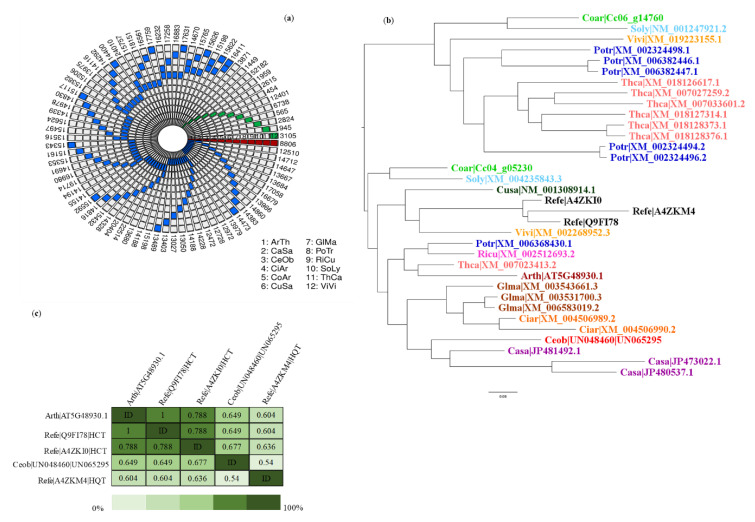
Unique genes and orthologs shared among the compared species (**a**). Each concentric circle corresponds to a species ((1) *Arabidopsis thaliana* (ArTh); (2) *Cannabis sativa* (CaSa); (3) *Cecropia obtusifolia* (CeOb); (4) *Cicer arietinum* (CiAr); (5) *Coffea arabica* (CoAr); (6) *Cucumis sativa* (CuSa); (7) *Glycine max* (GlMa); (8) *Populus trichocarpa* (PoTr); (9) *Ricinus communis* (RiCu); (10) *Solanum lycopersicum* (SoLy); (11) *Theobroma cacao* (ThCa); (12) *Vitis vinifera* (ViVi)). The blue blocks mark the intersections in pairs, the green blocks refer to the unique genes of each species, and the red blocks correspond to genes shared among all species compared. The number of orthologous genes is shown outside the concentric circles. To generate this figure and visualize the multispecies intersections, the R package SuperExactTest was used [41]. Phylogenetic reconstruction (**b**) represents the group of orthologous genes (orthogroup 776) identified as possibly encoding hydroxycinnamoyl-CoA quinate hydroxycinnamoyltransferase and hydroxycinnamoyl-CoA shikimate/quinate hydroxycinnamoyl transferase (HQT/HCT). The figure shows a rooted tree used as an external group plant species belonging to the group of asterids using the maximum likelihood (ML) model. (**c**) Identity matrix obtained after the phylogenetic reconstruction of the same orthogroup.

**Figure 8:**
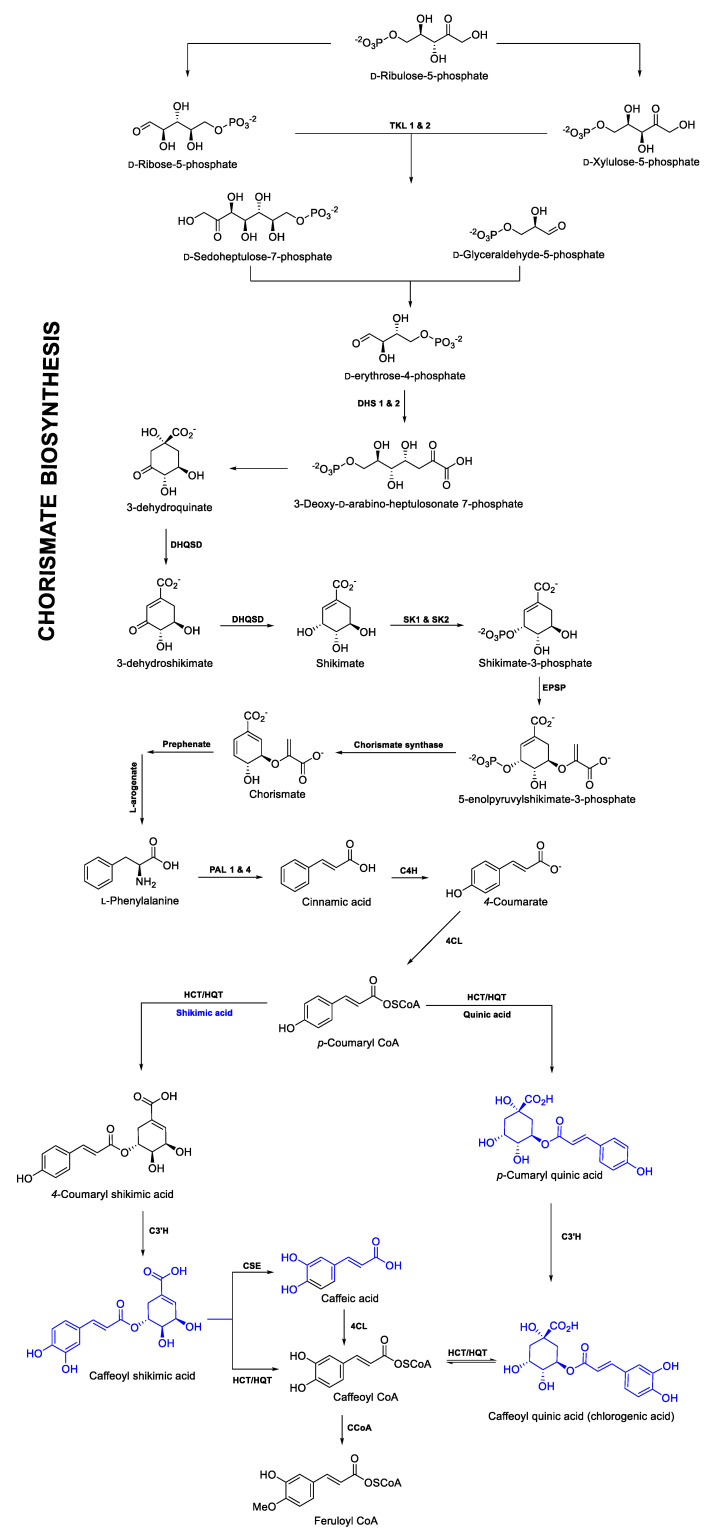
Biosynthesis of CGA in *C. obtusifolia* proposed from chemical-analytical and computational-theoretical analyses.

In addition to the homolog searches, an analysis was carried out with the OrthoMCL program to identify and group orthologs (putative) between the compared species shown in Supplementary Table S8, including *C. obtusifolia* (Figure 7a). A total of 17 orthogroups with a total of 54 unigenes of *C. obtusifolia* were identified and considered to be orthologs of reference enzymes involved in CGA biosynthesis are shown in Supplementary Table S9. These alignments for each orthogroup between all proteins were performed to reconstruct the corresponding phylogenetic tree, shown in Supplementary Materials Figure S5a–q (see as example Figure 7b). Generally, in most of the phylogenetic trees, the orthologous proteins were resolved as expected, considering their evolutionary history (Figure 4). According to the identity matrices shown in Supplementary Tables S10–S26, the similarities between the sets of orthologous enzymes are in the range of 40% to 70% (see example Figure 7c). It should be noted that in cases in which orthologous proteins were grouped into several sister clades, the unigenes of *C. obtusifolia* belonging to the clades containing the reference proteins were considered the primary candidates involved in the CGA biosynthesis pathway. Moreover, using RNA-seq data, the expression patterns of all the candidate genes involved in the CGA pathway were analyzed. The FPKM values obtained for each unigene were used for this purpose and they are shown in Supplementary Table S27 and Figure S6a–q. (1) phenylalanine ammonium lyase (PAL), (2) *trans*-cinnamate 4-monooxygenase (C4H), (3) *p*-coumaroyl quinate/shikimate 3′-hydroxylase (C3′H), (4) hydroxycinnamoyl-CoA quinate hydroxycinnamoyltransferase and hydroxycinnamoyl-coenzyme A shikimate/quinate hydroxycinnamoyl transferase (HQT/HCT), (5) tapetum specific methyltransferase 1 (CCoA), (6) chorismate synthase, (7) 4-coumarate-CoA ligase (4CL) and (8) caffeoyl shikimate esterase (CSE) were some of the enzymes involved in the route and showed considerable expression with FPKM values not lower than 50 (these values are shown in Supplementary Table S27 and Figure S6c,d,j–l,o–q). These genes are intimately related to the path of phenylpropanoids in higher plants, as well as in the biogenesis of hydroxycinnamic acids, such as ferulic acid, caffeic acid, CGA, among others, in which their obvious overexpression is due to abiotic stress, there were also genes for tapetum-specific methyltransferase 1, which synthesizes caffeoyl-CoA and feruloyl-CoA, and chorismate synthase, which is the enzyme responsible for producing prephenate, a compound precursor of L-arogenate and consequently of L-phenylalanine. Finally, the CGA biosynthesis pathway is described based on chemical-analytical and theoretical-computational analysis, which is consistent with previously reported results [38,39,40] (Figure 8).

## 3. Discussion

### 3.1. Evaluation of Cell Proliferation of Cell Cultures in C. obtusifolia Suspension

*C. obtusifolia* cell suspensions exhibited a logarithmic phase between seven and 28 days after the establishment of cell cultures under optimal nitrate conditions (27.4 mM) (Figure 1). When cell suspensions were established under moderately limiting nitrate conditions (16 and 8 mM), the logarithmic phase was similar to that under the control condition during the first 21 days. At the end of the kinetics time course (28 days), at both concentrations (16 and 8 mM), cellular proliferation was arrested, and the viability seemed to be compromised because of nitrate limitation (Figure 1). Regarding the 4 mM treatment, an increase in cellular proliferation was observed, but this increase was significantly less than that under the control treatment (27.4 mM). The loss of cellular viability observed was consistent with the presence of certain enriched functional categories in the list of *C. obtusifolia* unigenes. The categories identified as differentially expressed under the stress treatments (16 and 4 mM) by transcriptomic analysis included categories related to cell death, programmed cell death, and oxidative stress, where the number of genes was considerably higher for 16 mM treatment (in which a loss of viability was observed in terms of cell proliferation). These categories explain the cellular decay since reactive oxygen species (ROS) accumulate during the kinetics time course and could induce programmed cell death. Thus, autophagy could be one of the mechanisms that allows the recycling and remobilization of nutrients before an imminent collapse due to its capacity to degrade cellular content and decompose harmful or toxic material generated as a result of the accumulation of ROS [42,43,44].

### 3.2. Identification of CGA by Chemical-Analytical Analysis

Our results show that CGA biosynthesis increased throughout the kinetics time course. These results are consistent with those reported by [18], where the presence of this metabolite increased along a kinetics time course of 32 days established for *C. obtusifolia* under nitrate-limiting conditions. These results are also consistent with those reported in different plant species, such as *Morinda citrifolia* L. [45], *Cecropia peltata* L. [46], *Medicago truncatula* Gaertn. [47] and *Castilleja tenuiflora* Benth. [48], where nitrate restriction stress likewise induces CGA biosynthesis and other phenolic compounds. In the case of isoorientin (ISO), the results obtained in this study contrast with those reported by [18] in the same work, since in this study, no ISO was detected. The detection of CGA in chemical-analytical analysis is because this phenolic compound is a constitutive SMe, and in response to different types of stress (mainly abiotic), it does not require phenological states or an apparent compartmentalization for its biosynthesis [49,50,51].

### 3.3. Induction of CGA Biosynthesis in Nitrate Deficiency

A substantial and sustained increase in CGA accumulation was observed throughout the experiment. The measurements at 21 and 28 days showed the maximum point of CGA accumulation and represented an increase of more than 50% with respect to the other conditions, including the control (27.4 mM), see Supplementary Figure S2. These results provide confirmatory evidence that the increase depends directly on nitrate starvation stress. Co-chromatography carried out by enriching some of the samples with the commercial standard used as a reference provided evidence that the CGA detected in *C. obtusifolia* cell suspensions corresponds to the regio-isomer 3-CQA. This is consistent with many reports published in the literature, where it is reported that in the vast majority of plant species chlorogenic acid (3-CQA) is the most abundant regio-isomer among the caffeoylquinic acid derivatives. It is important to consider that according to the current International Union of Pure and Applied Chemistry (IUPAC) specifications, 3-CQA is nowadays called 5-caffeoylquinic acid (5-CQA). This is due to the fact that in 1976, the IUPAC reversed the order of numbering of the carbon atoms in the quinic acid ring and suggested chlorogenic acid (3-CQA) as 5-CQA, despite this, most of the commercial chemical suppliers still use the pre-IUPAC nomenclature. Therefore, in this work, as probably in many others previously published, CGA of *C. obtusifolia* corresponds actually to 5-CQA isomer considering the correct IUPAC name [52,53,54,55,56,57,58].

### 3.4. Description of the CGA Biosynthetic Route from Chemical-Analytical and Theoretical-Computational Analyses

Although CGA has been identified in different plant species, the complete biosynthesis pathway is still debated [53]. Three routes have been proposed so far [38,39,40,53,59,60,61] based on studies in dicotyledonous species such as *Solanum lycopersicum* L., *Nicotiana tabacum* L. and *Cynara cardunculus* L. [62]. Although HCT and HQT are homologous proteins with a high percentage of identity (> 80%), in some botanical taxa, these enzymes have been demonstrated to have a certain role. For example, HCT is related to lignin biosynthesis [63], and HQT directly participates in CGA biosynthesis, studies conducted in *Nicotiana benthamiana* Domin. demonstrated that the suppression of HQT decreases the CGA content by up to 98% [39,40]. However, in some plant species, such as *Arabidopsis thaliana* (L.) Heynh. there seems to be a single protein acting as a dual function HQT/HCT [AT5G48930] [64]. In the case of *C. obtusifolia*, a unique orthologous protein of HCT and HQT was identified, both enzymes were used as reference proteins and were placed in the same orthogroup with the corresponding ortholog in *Arabidopsis*. These data, together with the metabolites identified as possible precursors, suggest that the enzyme existing in *C. obtusifolia* performs a dual function. Its activity could be favored by the availability of certain substrates, such as *p*-cumaroyl-shikimic acid and shikimic acid caffeoyl [38,63]. The fact that HQT and HCT belong to the same orthogroup indicates that these enzymes are present in some species because of a gene duplication event or duplication of the complete genome (polyploidy). Two copies of a single ancestral gene were subfunctionalized to perform a single function in a specific way [65]. However, the enzyme present in species such as *Arabidopsis* or *C. obtusifolia* may be able to continue to play a bifunctional role, both favoring the synthesis of certain phenolic compounds such as CGA and promoting lignin biosynthesis. Other data that support this hypothesis include the fact that only one of the four unigenes (UN073057) identified in the transcriptome of *C. obtusifolia* was an ortholog of the PAL enzymes and showed a considerable increase in transcripts under nitrate deficiency treatments, mainly at the earliest time point (three hours) evaluated. According to the corresponding annotation, this unigene is homologous to the *Arabidopsis* PAL1 protein (AT2G37040).

Interestingly, a study in tobacco has shown that the expression of HQT and the orthologs of PAL seem to be related to a considerable increase (approximately three times higher) in CGA detected after independent overexpression of HQT and PAL [66]. The CGA increase was observed as a consequence of PAL1 overexpression, reported only for this member of the PAL family, and it has been suggested that the rest of the family members (PAL2, PAL3, and PAL4) could be involved in lignin synthesis [67], but these functions differ according to the organism studied. Consequently, it has been reported that in *Arabidopsis* species, the family coding for PAL proteins consists of five members, PAL1 and 2 are closely involved in the biosynthesis of phenylpropanoids, but PAL1 is also involved in lignin biosynthesis [68]. Poplar (*Populus trichocarpa* Torr. & A. Gray ex Hook.) contains five members of the PAL family, and it has been shown that PAL1 and PAL3 are strongly involved in the phenylpropanoid pathway [69], while PAL2, PAL4, and PAL5 are predominantly responsible for lignin production [70,71]. The above shows that, regardless of the number of PAL family members, there are members whose function seems to be more closely related to the synthesis of phenylpropanoids, as demonstrated in this study. Finally, the presence of the ortholog (UN086367) for the CSE enzyme (AT1G52760.1) stands out, its participation as a key enzyme in the biosynthetic pathway of lignin and phenylpropanoids is still debated because it is not yet clear if this catalytic step is preserved in most botanical lineages. However, it has been shown that this enzyme is involved in phenylpropanoid biosynthesis in *Arabidopsis thaliana*, *Medicago truncatulata* Godr. & Gren and *Populus × euramericana* Guinier because these botanical species all contain homologous CSEs, which have been identified either in their genome and/or transcriptome; in some cases, the amount of transcript and the corresponding enzyme have been positively related in lignifying tissues and the phenylpropanoid pathway [72,73,74,75,76]. This is consistent with the observations in this study, where *C. obtusifolia* also potentially houses this enzyme, which makes sense considering its high CGA biosynthesis in different organs of the plant.

**Figure 9:**
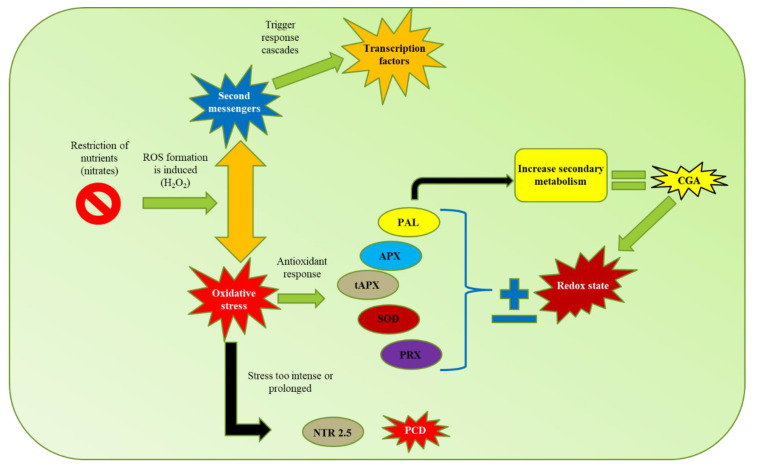
Proposed model of response mediated by abiotic stress (nitrate starvation) in *C. obtusifolia*. Compounds marked in blue were identified in the targeted and untargeted metabolomic analyses.

According to the results and the analyses carried out in this study, a model is proposed (Figure 9) of CGA biosynthesis in response to nitrate restriction in *C. obtusifolia*. This study suggests that nutrient stress induces reactive oxygen species production (especially H_2_O_2_), which functions as a second messenger, triggering a series of responses; moreover, prolonged exposure in the system induces oxidative stress. As a defense mechanism, the cells activate an “antioxidant” response to modify the redox state. As part of this response, the expression of specific genes is induced, such as PAL, which increases the biosynthesis of metabolites with antioxidant properties such as CGA, allocating nutritional resources to the functioning of secondary metabolism, compromising growth to promote survival. If the stress is too prolonged or severe, programmed cell death is activated to detoxify the cells and remobilize nutrients to avoid system collapse. This proposed model has a strong relationship with the carbon–nutrient balance (CNB) hypothesis, which postulates that the carbon-nutrient status of plants is determined by the availability of the source, and within the conceptual framework of this hypothesis, the SMe in the plant tissues are synthesized according to the relative abundances in the available resources. Environmental variation can influence the availability of certain resources necessary for plant growth, such as nitrogen and carbon fixed during photosynthesis, this variation can also directly affect and generate significant quantitative changes in the production of allelochemicals and other secondary metabolites used as defense.

## 4. Conclusions

In the present work, we performed an integrated study considering the transcriptome and metabolome of *C. obtusifolia* under different nitrate concentrations. We found the accumulation of different phenolic compounds involved in CGA biosynthesis and the upregulation of the genes involved in its metabolic pathway.

## 5. Materials and Methods

### 5.1. Callus and Cells in Suspension Cultures

According to previously reported methodology [18], in this study, we used young leaves of *Cecropia obtusifolia* Bertol. collected from an acclimatized plant in a demonstration plot (South Biomedical Research Center of the Mexican Institute of Social Security (CIBIS-IMSS), Xochitepec, Morelos, Mexico)), which were kindly provided by Dr. Maria del Pilar Nicasio-Torres. Later, they were disinfected using a soap solution and several rinses of 70% ethanol (3 min), 1.2% sodium hypochlorite solution and 0.2% Tween-20 (10 min); as the final step, the leaves were washed at least three times using sterile distilled water. Each disinfected leaf was cut into 5 mm^2^ sections and then transferred to glass vessels (250 mL) with 40 mL of 50% Murashige and Skoog (MS) culture medium supplemented with 15 g L^−1^ sucrose and 8 g L^−1^ agar, followed by filtration with 50 mg L^−1^ chloramphenicol (Sigma) and 4.5 mg L^−1^ amphotericin B (Sigma). The explants were incubated at 26 ± 2 °C during a light:dark (16:8-h) photoperiod under 32 μmol m^−2^ s^−1^ [77].

Once cell cultures were established, and after waiting one week, the non-contaminated leaf explants were transferred to MS culture medium supplemented with 30 g L^−1^ sucrose, 2 mg L^−1^ 2,4-dichlorophenoxyacetic acid (2,4-D), 0.5 mg L^−1^ of benzylaminopurine (BAP) and 8.0 g L^−1^ of agar and pH adjusted to 5.8, as already reported as the best complements for callus development. The explants were incubated at conditions as described above and subsequently transferred to a new medium every 4 weeks until callus was obtained (Nicasio-Torres et al., 2012). The suspension cell culture of *C. obtusifolia* was started by transferring 6 g of fresh biomass corresponding to the generated callus, in 80 mL of liquid MS medium with sucrose 30 g L^−1^, 2,4-D, 2 mg L^−1^ and BAP, 0.5 mg L^−1^, see Supplementary Tables S28 and S29. The cultures were placed in a rotatory shaker at 110 rpm (New Brunswick Scientific Co.), employing the same culture conditions described previously. The cell biomass was filtered under sterile conditions and cultured in the same fresh MS medium every 2 weeks and culture conditions over a period of 5 months until enough biomass was obtained to establish the kinetic temporary courses [18].

### 5.2. Temporal Kinetics of Cells in Suspension

CGA growth kinetics and production consisted of evaluating three independent biological replicas at five time points after the establishment of stress, either nitrate deficiency or nitrate starvation: time 0 (T0; corresponding to the initial culture used from which the inoculate was taken, evaluated before the transfer to the corresponding MS culture medium with reduced nitrates), T1 (3 h after the transfer at T0 to new culture medium), T2 (7 days), T3 (14 days), T4 (21 days) and T5 (28 days). We used 27.4 mM nitrate as a control and, as nitrate starvation, three different concentrations of total nitrates (16, 8 and 4 mM potassium/ammonium nitrate) in the culture medium. The biomass contained in each of the flasks used in the kinetic was filtered, using a vacuum pump (BUCHI Vacuum Pump V-700) and filter paper (Whatman, PWDF 0.45 μm) in a Kitasato flask until the excess MS medium was eliminated. Subsequently, the biomass was fractionated. For the RNA extraction process, 300 mg of fresh biomass was taken from the total and placed in a 1.5 mL tube, which was frozen with the help of liquid nitrogen (−186 °C) and stored at −80 °C for later use. The remaining biomass was used in the high-performance liquid chromatography (HPLC) analysis.

### 5.3. Quantitative Analysis of Methanolic Extracts by HPLC

The remaining biomass, once the required amount was taken for RNA isolation, was taken to dry for 24 h (THELCO Laboratory oven) at 65 °C, and then extractions were carried out with methanol (1:20 g L^−1^) at room temperature for 24 h. Three replicates were processed per sample. After filtering and collecting the extracts obtained from the biomass, they were taken to dryness by reduced pressure and were recovered in 10 mL of HPLC grade methanol, and subsequently analyzed. HPLC analysis was performed in a Waters 996 device with a binary pump (2695) coupled to a diode array detector (2696) with a detection range of 190 to 600 nm operated by the Millenium System Manager Software system (Empower 1), using a Spherisorb-ODS RP-18 column (250 × 4.6 mm, 5 μm, Waters) at 25 °C, chromatographic separation was performed using a gradient elution system with A = acidulated water (CF_3_-COOH, 0.5% trifluoroacetic acid (TFA)) and B = CH_3_CN, maintaining a constant flow of 1.2 mL min^−1^ according to the following scheme: 2 min = 100% A; at minute 3, 95% of A; at minute 7, 70% of A; at minute 12, 50% of A; at minute 17, 20% of A; at minute 21, 0% of A; and finally at minute 24, 100% of A, keeping it for 1 min. The evaluation of the samples was performed with commercial standards with calibration curves of 10, 20, 40, 80 and 160 μg ml^−1^ for the CGA (Sigma-Aldrich; (Cat C3878:) and 5, 10, 20, 40 and 80 μg mL^−1^ for ISO (Sigma-Aldrich), with wavelengths of λ = 327 for CGA and the retention time was 13,299 min. The kinetic growth constants were calculated, and finally, statistical analysis was performed with Number Cruncher Statistical Software (NCSS) software version 5 using a factorial analysis of variance (ANOVA) followed by Tukey’s range test. Values of *p* ≤ 0.05 were considered statistically significant.

### 5.4. Quantitative Analysis of Phenolic Compounds by Targeted Metabolomics Approach

The phenolic targeted metabolomics with methanolic extracts analysis was performed as described by [78]. An Agilent 1290 ultrahigh-resolution liquid chromatograph (UPLC) coupled to a triple quadrupole mass spectrometer (QqQ, Agilent 6460) with a dynamic multiple reaction monitoring (dMRM) method with 60 authentic standards was used. The mobile phases consisted of water with 0.1% formic acid (A) and 90% of acetonitrile (aqueous solution) with 0.1% formic acid (B), both MS grade (Sigma), and the flow was 0.1 mL min^−1^ (see Supplementary Table S30). The gradient conditions for the mobile phase were as follows: 0 min 1% B, 0.1–40 min linear gradient 1–40% B, 40.1–42 min linear gradient 40–90% B, 42.1–44 min isocratic 90% B, 44.1–46 min linear gradient 90–1% B, 46.1–47 min 1% B isocratic. The injection volume was 1 μL, and the column used was an Agilent, Zorbax SB-C18, 2.1 × 50 mm, 1.8 Microns. The column temperature was 40 ± 0.8 °C. The mass spectrometry conditions of the dMRM method are shown in Supplementary Materials Table S31. Phenolic compounds quantification was performed with calibration curves in a concentration range 0.5–17 μM established for each compound; in some cases, we performed sample dilutions to fit to a standard curve, see Supplementary Table S32.

### 5.5. Identification of Chemical Markers by Untargeted Metabolomics Analysis

To identify SMe as differential markers between treatments, methanolic extracts were previously obtained, 500 μL of each sample was taken and placed in 1.5 mL tubes, and then 5 μL of formic acid at 0.1% was added to each sample as an ionizing agent. Finally, 300 μL of each sample was placed in 1 mL glass vials for the comparative analysis of metabolic profiles using a Waters class I UPLC coupled to a high-resolution mass spectrometry Waters Synapt G2-Si quadrupole-time-of-flight (QTOF). The discriminative criteria for the selection of SMe identified through the Metabolite and Chemical Entity Database (METLIN) [79] were the following: (i) those metabolites that were analyzed and plotted (S-plot) further away from the X and Y axis were selected, since they are the ones that contribute most to differentiate the treatments that are contrasted; (ii) only those SMe that would yield an identification were selected when comparing them in the database; (iii) only SMe were chosen that were represented in plants and that had interference in the biosynthetic route of the compound of interest. The conditions of analysis were the following: the mass spectrometric analysis was performed with Waters class I UPLC coupled to a high-resolution mass spectrometry Waters Synapt G2-Si quadrupole-time-of-flight (QTOF). Chromatography was carried out on an Acquity ethylene bridged hybrid (BEH) column (1.7 µm, 2.1 × 50 mm) with a column and sample temperature of 40 °C and 15 °C, respectively. The mobile phase consisted of (A) water and (B) acetonitrile, both with 0.1% of formic acid (SIGMA). The gradient conditions of the mobile phases were 0–13 min linear gradient 1%–80% B, 13–14 min 80% B isocratic, 14–15 min linear gradient 80%–1% B (total run time 20 min). The flow rate was 0.3 mL min^−1^ and 1 μL of the extract was injected. The mass spectrometry analysis was performed with an electrospray ionization source in negative and positive mode. The capillary voltages of the sampling cone and source compensation were 3000, 40 and 80 V, respectively. The source temperature was 100 °C and the desolvation temperature was 20 °C. The desolvation gas flow was 600 L h^−1^ and the nebulizer pressure was 6.5 Bars. Leucine-enkephalin was used as an internal calibrator (556.2771, [M + H]^+^, 554.2615, [M-H]^−^). The conditions used for the SMe analysis were mass range 50–1200 Da, function 1 CE, 6 V, function 2 CER 10–30 V, exploration time 0.5 s. The data were acquired and processed with the MassLynx (version 4.1) and MarkerLynx (version 4.1) software. A discriminatory analysis of orthogonal partial least squares (OPLS-DA) was carried out in order to identify specific biomarkers of the different treatments. Retention times and mass/charge ratios were considered with a noise threshold of 10,000 accounts. The Pareto scale was used to generate the scoring plots; and, finally, in the obtained graphs (S-plots), the variables that contributed to the discrimination between two analyzed groups were considered differential chemical biomarkers between conditions [80].

### 5.6. De Novo Transcriptome Assembly

For RNA isolation, library preparation and sequencing, three independent replicates per analyzed condition were used, corresponding to concentrations of 27.4 mM (control), 16 and 4 mM (stress induced by nitrate deficiency), with time points of 3 h, 7, 14, 21, and 28 days, resulting in a total of 45 samples being processed (Supplementary Table S32). Zero time (considered the beginning of the experiment) was eliminated from the comparison because 3 h after transferring the cells to the corresponding stress conditions, no significant differences were observed in terms of cell proliferation (for more information, see results section) at any of the tested concentrations.

Of the total biomass generated, 300 mg was fractionated, and only 100 mg of fractionated biomass per sample was used for total RNA isolation, using the Plant RNA Purification Reagent Kit (Thermo Fisher Scientific), following the manufacturer’s instructions. The RNA concentration was determined with a BioSpec-nano UV-VIS spectrophotometer (Shimadzu) with absorbances at 260 nm [81,82]. The integrity of the RNA was evaluated on a 1% nondenaturing agarose gel with the Invitrogen™ 1Kb Plus DNA Kit (Invitrogen) and analyzed with the BIORAD Gel DocTM EZ Imager with ImageLab Sol build 8. Approximately 3.5 μg of the total RNA isolated per sample was used to generate 45 libraries, for which the TruSeq RNA Sample Preparation v2 Kit (Illumina) was used following the manufacturer’s instructions, and index codes were added to identify each sample independently. The quality of the libraries sequenced was evaluated by capillary electrophoresis with a Bioanalyzer 2100 (Agilent Technologies). Each cDNA library was prepared at a final concentration of 20 mM and sequenced using the NextSeq500 (Illumina) with the 2 × 150 bp paired-end sequencing protocol format. All sequencing data have been deposited in NCBI Sequence Read Archive (SRA) under accession no. PRJNA594936.

Subsequently, the transcriptome assembly was carried out. First, the raw data in fastq format were filtered to eliminate low-quality sequences using a python script, available from GitHub [83]. The parameters used for the selection of high-quality (HQ) paired-end reads were -q 30 (minimum quality score to keep), -p 90 (minimum percent of bases in the sequence that must have a [–q] quality) and -a 28 (the minimum average quality). Then, the SeqPrep program [84] with the options -L 150 (minimum reading length) and -o 25 (minimum overlap of bases to join two readings) was used to identify overlapping regions at the ends of reads R1 and R2 and to remove adapter remnants and orphan reads.

Trinity assembler [85] was used to carry out the de novo assembly using the paired-end sequences that passed the quality filters, including the long sequences resulting from the union of paired-end readings that showed overlapping regions. The Trinity program was executed using the default parameters in which all the generated data (45 libraries) were combined. The resulting contigs, called unique transcripts or unigenes, were processed with the SeqClean program [86] to trim and eliminate terminal regions of low complexity and rich in undetermined bases (N’s) or poly A/T tails from the sequences. As a precautionary measure to identify and eliminate the existence of possible contaminating sequences, the DeconSeq program [87] was used. It was expected that the unigenes resulting from the assembly process would be free of contaminants because the cell cultures were established under aseptic conditions. Only the unigenes processed according to the previously described process and whose sequences were greater than or equal to 200 bp were considered in subsequent steps of the analysis.

### 5.7. Identification and Annotation of Protein Coding Regions

To generate a nonredundant data set representative of the *C. obtusifolia* transcriptome, coding regions were identified in the unigene sequences, and the erroneous reading frameshifts were corrected. These framing errors caused by insertions or deletions and resulting from the assembly process were identified by the alignment of each assembled unigene against homologs present in a “homemade” database of coding sequences and their corresponding proteins from approximately 100 plant species whose genomes have been fully sequenced (latest versions available in GenBank for selected angiosperm plant species). For this purpose, we used the AlignWise pipeline [19], an algorithm that, in an orderly, recursive and systematic way, handles different programs such as the Basic Local Alignment Search Tool (BLAST) [88] for the identification of homologous sequences with a high percentage of similarity; multiple sequence alignment by log-expectation (MUSCLE) [89] to align multiple sequences; and GeneWise [90], which identifies the coding regions and corrects erroneous frameshifts. Subsequently, the BLASTClust program [91] was used to create a set of nonredundant sequences. Coding sequences derived from unigenes with a nucleotide level that showed 95% identity along a region representing at least 90% of the length of another unigene were eliminated to avoid redundancies.

Using the best BLAST hit method (BBH), homologs to *C. obtusifolia* proteins were identified using an E-value threshold ≤ 10^−5^. Twelve different species of angiosperm plants belonging to specific lineages in the eudicotyledonous class were used as references. Species such *Cannabis sativa*, *Prunus persica*, *Cucumis sativa*, *Ricinus communis*, *Populus trichocarpa*, *Cicer arietinum*, *Glycine max*, *Theobroma cacao*, *Arabidopsis thaliana*, and *Vitis vinifera* were included as members of the asterid monophyletic group, while *Solanum lycopersicum* and *Coffea arabica* were included as an external group (members of the rosid clade). This group of plant species was selected because they have been reported to contain high amounts of CGA. The proteomes of these species represent the total of predicted gene models in their genome, except *Cannabis sativa*, for which the proteins used as references came from available transcriptomic data. All these sequences were obtained from the latest versions available in GenBank [92]. In addition to the search for homologous proteins identified in the genome of other plant species, the process of annotating the unigene set of *C. obtusifolia* also included the assignment of Gene Ontology terms [23] (GO terms) [24]. The unigenes of *C. obtusifolia* inherited these terms from the homologous proteins identified in *Arabidopsis thaliana*.

### 5.8. Expression Profiles and Identification of Differentially Expressed Genes (DEGs)

The genes that responded to nitrate starvation were identified by comparing the overall expression profiles from each set of transcriptomic data generated and analyzed over time (3 h, 7, 14, 21 and 28 days). RSEM software was used [20] because it uses short reading mappers, such as Bowtie2 [93], to independently assign the readings generated for each condition (different concentrations of nitrates at each sampling point) to each unigene resulting from the process assembly. The number of readings assigned to a single unigene represents the relative abundance of mRNA produced by a particular gene in a given sample. The expectation maximization algorithm implemented in RSEM produces a matrix of expression profiles where transcript abundance is represented by the “expected counts” and their normalized values, transcripts per million (“TPM”) and fragments by kilobases of contigs/unigenes per million mapped readings (“FPKM”) (Supplementary Table S6). The expected reading counts estimated by RSEM were used as input for DESeq [21], a package of R/Bioconductor [22] that normalizes samples by pairwise comparison and uses the relative logarithmic expression method to adjust the relationship values of the gene expression levels in relation to the initial value, given the hypothesis that most genes are not differentially expressed. After pairwise sample comparisons (27.4/16 mM, 27.4/4 mM, and 16/4 mM), a threshold was selected to identify differentially expressed unigenes, an adjusted probability value (adjusted *p*-value) ≤ 0.01 in a level confidence interval (1 − α), that is, a minimum confidence level of 99%. Finally, with the enrichment analysis of GO functional categories performed after the annotation process, it was possible to obtain information regarding the biological processes involving the unigenes of *C. obtusifolia* differentially expressed in response to stress due to nitrate starvation. This ontological-functional enrichment allowed us to identify processes that are affected in response to the stimulus and their importance in the adaptation of the organism.

### 5.9. Identification of Orthologous Genes

Orthologous and paralogous genes were identified using the OrthoMCL program [94] and the Markov clustering algorithm (MCL) with an inflation value of 1.5 [95]. To avoid most false positive results, a minimum entry length of 30 amino acids was considered in all the proteins used in the annotation analysis, and the threshold used for bidirectional BLAST (all vs all) was an E-value of < 10^−10^. The proteomes included in the orthology analysis were supplemented by adding sequences of 15 proteins from different plant species (Supplementary Table S33). These enzymes were considered “reference enzymes” because all of them have been functionally characterized, and their participation in the CGA biosynthetic pathway has been demonstrated (MetaCyc database) [96].

### 5.10. Phylogenetic Trees

Alignments and phylogenetic relationships between the proteins identified as orthologs of different plant species were grouped with the “reference enzymes” known to be involved in CGA synthesis and solved with the SeaView program [97]. MUSCLE [89] and PhyML v3.0 [98] were used for the alignments and phylogenetic analysis. The PhyML option was used under the Le and Gascuel (LG) model. To define the initial topology of the trees, in all cases, the distance method bio-neighbor-joining (BIoNJ) was used, while equilibrium frequencies, topologies, and branch length were optimized. The representative tree was selected after comparing the trees generated by the nearest-neighbor interchange (NNI) method and by the subtree pruning and regrafting (SPR) method. Finally, the support value of the branches was estimated using the approximate likelihood-ratio test (aLRT) [99].

## Figures and Tables

**Table 1:** Kinetic constants of cell suspension cultures of *C. obtusifolia* at different concentrations of nitrates in MS medium.

Condition NO_3_ (mM)	Average Maximal Biomass in Dry Weight (g L ^−1^)	Duplication Time (Days)	µ (Days^−1^)
27.4 mM	0.433 ^a^	32.83 ^a^	0.021 ^a^
16 mM	0.426 ^a^	25.33 ^a^	0.028 ^a^
8 mM	0.433 ^a^	30.18 ^a^	0.023 ^a^
4 mM	0.313 ^a^	65.72 ^b^	0.011 ^b^

Statistical significance is indicated by different letters (a, b) as assessed by Tukey’s test.

**Table 2:** Phenolic compound dynamics obtained in the temporary kinetic course of the cell cultures in suspension of *C. obtusifolia*. The concentration is expressed in µg g^−1^ dry weight.

Compounds			Nitrates (mM)	Time				
				3 Hours	7 Days	14 Days	21 Days	28 Days
**Phenylpropanoid pathway**	**Upstream**	**Shikimic acid**	27.4	ND	ND	ND	ND	ND
			16	ND	6.4 ± 5.6	20.3 ± 0.6	17.4 ± 5.4	27.4 ± 15.7
			4	ND	ND	14.3 ± 6.8	26.9 ± 23.9	20.6 ± 4.2
		*t*-Cinnamic acid	27.4	ND	ND	ND	ND	ND
			16	ND	ND	ND	3.9 ± 0.8	2.2 ± 2.1
			4	ND	ND	ND	ND	ND
		*p*-coumaric acid	27.4	ND	ND	ND	ND	2.2 ± 1.2
			16	ND	ND	ND	ND	ND
			4	7.3 ± 7.9	7.2 ± 11.1	ND	ND	ND
		Caffeic acid	27.4	89.6 ± 8.7	82.1 ± 15.9	63.0 ± 8.1	59.3 ± 2.3	53.0 ± 5.7
			16	93.4 ± 23.3	93.6 ± 4.3	76.7 ± 6.1	250.9 ± 5.4	345.6 ± 50.7
			4	84.2 ± 5.5	88.5 ± 18.2	86.4 ± 2.7	102.3 ± 45.5	85.5 ± 10.8
	Chlorogenic acid		27.4	69,437.7 ± 13,071.5	65,014.6 ± 7730.3	46,218.1 ± 7753.6	50,271.1 ± 872.4	50,515.6 ± 8999.8
			16	73,933.2 ± 26,317.3	62,615.2 ± 9428.4	60,899.9 ± 7435.3	82,379.5 ± 13,363.7	102,392.2 ± 10,253.0
			4	61,346.7 ± 4250.8	57,679.7 ± 7739.2	59,133.0 ± 23,575.2	75,282.7 ± 22,141.7	67,328.4 ± 21,360.3
	**Downstream**	Ferulic acid	27.4	ND	4.6 ± 2.1	7.5 ± 0.5	7.9 ± 1.8	8.9 ± 0.8
			16	ND	8.1 ± 5.9	8.2 ± 2.1	8.4 ± 1.7	8.1 ± 0.5
			4	4.4 ± 1.4	7.4 ± 6.5	9.5 ± 1.4	8.2 ± 3.5	6.9 ± 1.0
		Vanillin	27.4	22.7 ± 29.5	8.5 ± 1.8	3.0 ± 0.6	4.9 ± 0.3	19.1 ± 12.2
			16	9.8 ± 3.6	12.5 ± 10.9	4.7 ± 1.9	25.7 ± 18.2	26.6 ± 15.1
			4	43.8 ± 37.1	42.0 ± 56.7	5.9 ± 0.6	8.1 ± 4.4	10.3 ± 2.1
		Vanillic acid	27.4	36.5 ± 58.4	10.2 ± 4.8	ND	ND	17.9 ± 14.0
			16	ND	18.2 ± 17.8	3.5 ± 1.2	18.7 ± 15.7	18.2 ± 16.6
			4	39.3 ± 32.8	51.4 ± 79.7	ND	5.7 ± 7.5	4.1 ± 1.8
		Quercetin-3-d-galactoside	27.4	ND	17.77 ± 4.81	20.80 ± 0.21	19.19 ± 2.71	21.12 ± 5.69
			16	25.23 ± 11.46	39.77 ± 6.48	43.63 ± 1.44	55.97 ± 17.80	66.66 ± 13.73
			4	23.00 ± 3.73	29.36 ± 12.93	40.52 ± 6.93	42.13 ± 12.22	54.88 ± 17.37

ND: not detected.

## Supplementary Materials

Supplementary materials can be found at https://www.mdpi.com/1422-0067/21/20/7572/s1. Figure S1: Chromatographic profiles and absorption spectra of commercial standard of CGA and methanolic extract of suspension cell cultures (T21). Figure S2: Levels of CGA accumulation in relation to the reduction of total nitrates, in suspension cell cultures of *C. obtusifolia* along the kinetic time course. Figure S3: Chromatographic profiles and absorption spectra of methanolic extract (T21), and the same sample point enrichment with the commercial standard of CGA. Figure S4: Main secondary metabolites identified by mass spectrometry in both metabolomic analyses. Figure S5: Phylogenetic reconstruction for the group of orthologs genes identified as possible enzymes involved in CGA pathway. Figure S6: Levels of expression in FPKM for the orthologs gene group identified in the transcriptome of *C. obtusifolia*. Table S1: Abundance profile and standard deviation of secondary metabolites identified from methanolic extracts. Table S2: Tentative identification of phenolic compounds based on their fingerprints by mass spectrometry analysis. Table S3: Summary of sequencing data generated from cell cultures in suspension of *C. obtusifolia*. Table S4: Collection of representative transcripts of *C. obtusifolia*. Table S5: Functional categorization of *C. obtusifolia*. Table S6: Matrix expression profile of RNA-seq libraries with the expression values obtained using the Bowtie2 program. Table S7: Differential expression analysis performed using the DESeq package. Table S8: Analysis to identify orthologs made by comparing different botanical species capable of synthesizing CGA. Table S9: Orthologs genes involved in the biosynthesis of chlorogenic acid (CGA). Table S10: Identity matrix of orthogroup 7162, corresponding to the orthologous enzyme RPI2 (probable ribose-5-phosphate isomerase 2). Table S11: Identity matrix of orthogroup 8244 corresponding to the orthologous enzyme RPE (D-ribulose-5-phosphate 3-epimerase). Table S12: Orthogroup 1804 identity matrix corresponding to the orthologous enzyme TKL-1, TKL-2 (Transketolase 1 & 2). Table S13: Identity matrix of orthogroup 639 corresponding to the orthologous enzyme DHS 1 and 2 (phospho-2-dehydro-3-deoxyheptonate aldolase 1 & 2). Table S14: Identity matrix of orthogroup 7791 corresponding to the orthologous enzyme DHQS (3-dehydroquinate synthase). Table S15: Orthogroup 694 identity matrix corresponding to the orthologous enzyme DHQSD (3-dehydroquinate dehydratase/shikimate dehydrogenase). Table S16: Identity matrix of orthogroup 2332 corresponding to the orthologous enzyme SK-1 (shikimate kinase 1). Table S17: Identity matrix of orthogroup 5166 corresponding to the orthologous enzyme EPSP (3-phosphoshikimate 1-carboxyvinyltransferase). Table S18: Orthogroup 2732 identity matrix corresponding to the orthologous enzyme Chorismate synthase. Table S19: Orthogroup 205 identity matrix corresponding to the PAL orthologous enzyme (phenylalanine ammonia lyase). Table S20: Orthogroup 851 identity matrix corresponding to the orthologous enzyme C4H (Trans-cinnamate 4-monooxygenase). Table S21: Identity matrix of orthogroup 776 corresponding to the orthologous enzyme HQT/HCT (hydroxycinnamoyl-CoA quinate hydroxycinnamoyl transferase & hydroxycinnamoyl-CoA shikimate/quinate hydroxycinnamoyl transferase). Table S22: Identity matrix of orthogroup 1085 corresponding to the orthologous enzyme C3′H (p-coumaroyl quinate/shikimate 3′-hydroxylase). Table S23: Identity matrix of orthogroup 2776 corresponding to the orthologous enzyme CCoA (probable caffeoyl-CoA O-methyltransferase). Table S24: Identity matrix of orthogroup 2935 corresponding to the orthologous enzyme CCoA (tapetum specific methyltransferase 1). Table S25: Identity matrix of orthogroup 396 corresponding to the orthologous enzyme 4CL (4-coumarate-CoA ligase). Table S26: Identity matrix of orthogroup 11033 corresponding to the orthologous enzyme CSE (caffeoyl shikimate esterase). Table S27: Matrix of the expression profiles of the RNA-seq libraries. The expression values were obtained using the Bowtie2 program. Table S28: Concentrations of culture medium of Murashige and Skoog (MS). Table S29: Supplements used the culture medium of Murashige and Skoog (MS). Table S30: Chromatographic conditions for Agilent ultrahigh resolution liquid chromatograph. Table S31: Conditions of the Agilent 6460 mass spectrometer. Table S32: Analysis conditions and quantification ranges for the 60 standard compounds used to quantify phenolic compounds of interest. Table S33: Reference enzymes included in the orthology analysis.
