# Supplementary material for: Integrated Analysis of the Transcriptome and Metabolome of Cecropia obtusifolia: A Plant with High Chlorogenic Acid Content Traditionally Used to Treat Diabetes Mellitus
Source: Int J Mol Sci. 2020 Oct 14;21(20):7572. doi: 10.3390/ijms21207572 (PMC7588936; doi:10.3390/ijms21207572)
Supplement: Supplementary file 1 [file ijms-21-07572-s001.zip › Supplementary Material/Supplementary Figures.docx]

The following supplemental materials are available for the article entitled: "Integrated Analysis of the Transcriptome and Metabolome of *Cecropia obtusifolia*: A Plant with High Chlorogenic Acid Content Traditionally Used to Treat Diabetes Mellitus". The tables S1-S33 are in the excel file corresponding to the supplementary materials.

**Figure S1:** Chromatographic profiles and absorption spectra of commercial standard of CGA and methanolic extract of suspension cell cultures (T21).

**Figure S2:** Levels of CGA accumulation in relation to the reduction of total nitrates, in suspension cell cultures of *C. obtusifolia* along the kinetic time course.

**Figure S3:** Chromatographic profiles and absorption spectra of methanolic extract (T21), and the same sample point enrichment with the commercial standard of CGA

**Figure S4:** Main secondary metabolites identified by mass spectrometry in both metabolomic analyses.

**Figure S5:** Phylogenetic reconstruction for the group of orthologs genes identified as possible enzymes involved in CGA pathway.

**Figure S6:** Levels of expression in FPKM for the orthologs gene group identified in the transcriptome of *C. obtusifolia*.

**Table S1:** Abundance profile and standard deviation of secondary metabolites identified from methanolic extracts.

**Table S2:** Tentative identification of phenolic compounds based on their fingerprints by mass spectrometry analysis.

**Table S3:** Summary of sequencing data generated from cell cultures in suspension of *C. obtusifolia*.

**Table S4:** Collection of representative transcripts of *C. obtusifolia*.

**Table S5:** Functional categorization of *C. obtusifolia*.

**Table S6:** Matrix expression profile of RNA-seq libraries with the expression values obtained using the Bowtie2 program.

**Table S7:** Differential expression analysis performed using the DESeq package.

**Table S8:** Analysis to identify orthologs made by comparing different botanical species capable of synthesizing CGA.

**Table S9:** Orthologs genes involved in the biosynthesis of chlorogenic acid (CGA).

**Table S10:** Identity matrix of orthogroup 7162, corresponding to the orthologous enzyme RPI2 (probable ribose-5-phosphate isomerase 2).

**Table S11:** Identity matrix of orthogroup 8244 corresponding to the orthologous enzyme RPE (D-ribulose-5-phosphate 3-epimerase).

**Table S12:** Orthogroup 1804 identity matrix corresponding to the orthologous enzyme TKL-1, TKL-2 (Transketolase 1 & 2).

**Table S13:** Identity matrix of orthogroup 639 corresponding to the orthologous enzyme DHS 1 and 2 (phospho-2-dehydro-3-deoxyheptonate aldolase 1 & 2).

**Table S14:** Identity matrix of orthogroup 7791 corresponding to the orthologous enzyme DHQS (3-dehydroquinate synthase).

**Table S15:** Orthogroup 694 identity matrix corresponding to the orthologous enzyme DHQSD (3-dehydroquinate dehydratase/shikimate dehydrogenase).

**Table S16:** Identity matrix of orthogroup 2332 corresponding to the orthologous enzyme SK-1 (shikimate kinase 1).

**Table S17:** Identity matrix of orthogroup 5166 corresponding to the orthologous enzyme EPSP (3-phosphoshikimate 1-carboxyvinyltransferase).

**Table S18:** Orthogroup 2732 identity matrix corresponding to the orthologous enzyme Chorismate synthase.

**Table S19:** Orthogroup 205 identity matrix corresponding to the PAL orthologous enzyme (phenylalanine ammonia lyase).

**Table S20:** Orthogroup 851 identity matrix corresponding to the orthologous enzyme C4H (Trans-cinnamate 4-monooxygenase).

**Table S21:** Identity matrix of orthogroup 776 corresponding to the orthologous enzyme HQT/HCT (hydroxycinnamoyl-CoA quinate hydroxycinnamoyl transferase & hydroxycinnamoyl-CoA shikimate/quinate hydroxycinnamoyl transferase).

**Table S22:** Identity matrix of orthogroup 1085 corresponding to the orthologous enzyme C3'H (*p-*coumaroyl quinate/shikimate 3'-hydroxylase).

**Table S23:** Identity matrix of orthogroup 2776 corresponding to the orthologous enzyme CCoA (probable caffeoyl-CoA O-methyltransferase).

**Table S24:** Identity matrix of orthogroup 2935 corresponding to the orthologous enzyme CCoA (tapetum specific methyltransferase 1).

**Table S25:** Identity matrix of orthogroup 396 corresponding to the orthologous enzyme 4CL (4-coumarate-CoA ligase)).

**Table S26:** Identity matrix of orthogroup 11033 corresponding to the orthologous enzyme CSE (caffeoyl shikimate esterase).

**Table S27:** Matrix of the expression profiles of the RNA-seq libraries. The expression values were obtained using the Bowtie2 program.

**Table S28:** Concentrations of culture medium of Murashige and Skoog (MS).

**Table S29:** Supplements used the culture medium of Murashige and Skoog (MS).

**Table S30:** Chromatographic conditions for Agilent ultrahigh resolution liquid chromatograph.

**Table S31:** Conditions of the Agilent 6460 mass spectrometer.

**Table S32:** Analysis conditions and quantification ranges for the 60 standard compounds used to quantify phenolic compounds of interest.

**Table S33:** Reference enzymes included in the orthology analysis.

**Figure S1****.** Chromatographic profile (left) and absorption spectrum (λ = 327 nm) (right) obtained for (a) commercial standard chlorogenic acid, with a retention time of 13.299 min, and (b) chlorogenic acid from a methanolic extract generated from the cellular suspension of *C. obtusifolia* grown for 21 days at a concentration of 16 mM nitrates, with a retention time of 13.454 min.

**Figure S2** Levels of CGA accumulation in relation to the reduction of total nitrates and with elapsed time calculated for the suspension cell cultures of *C. obtusifolia*. The bars represent the standard deviation calculated from three biological replicates. The lowercase letters represent the ANOVA and Tukey’s clustering performed for each of the nitrate concentrations over time. In all nitrate concentrations evaluated throughout the kinetics time course, statistically significant differences were found, with increasing prominence at later times: 27.4 mM (F_0.05_ = 8.75, p = 0.003); 16 mM (F_0.05_ = 27.24, p = 0.001); 8 mM (F_0.05_ = 11.71, p = 0.001) and 4 mM (F_0.05_ = 32.77, p = 0.001).

**Figure S3** Chromatographic profile (left) and absorption spectrum (right) of chlorogenic acid (λ = 327 nm) with a retention time of 13.454 min. (a) Methanolic extract generated from the cellular suspension of *C. obtusifolia* grown for 21 d at a concentration of 16 mM nitrates. (b) Chromatographic profile and absorption spectrum generated for the same sample after enrichment with the commercial standard with a retention time of 13.137 min.

**Figure S4.** Major secondary metabolites identified by mass spectrometry that presented considerable quantification for at least two of the three concentrations of nitrates along the kinetic time courses. The bar graphs show the quantities detected in the targeted metabolomic study. These data shown the metabolites (listed below) that were identified in both metabolomic analyses performed in this study (metabolomics analysis). Chlorogenic acid (a), shikimic acid (b), *t*-Cinnamic acid (c), *p*-Coumaric acid (d), caffeic acid (e), ferulic acid (f), vanillic acid (g), quercetin-3-D-galactoside (h), vanillin (i), quercetin-3-glucoside (j), (+)-catechin (k), (-)-epicatechin (l), quercetin-3,4'-di-O-glucoside (m) and, 4-hydroxyphenylacetic acid (n).

**Figure S5.** Phylogenetic reconstruction for the group of orthologous genes identified as possible enzymes, where the species of interest *C. obtusifolia* is shown in red, and the sequences of the reference enzymes are shown in black. (a) orthogroup 7162, corresponding to Probable ribose-5-phosphate isomerase 2 [RPI2], (b) orthogroup 8244, corresponding to D-ribulose-5-phosphate 3-epimerase [RPE], (c) orthogroup 1804, corresponding to transketolase 1 & 2 [TKL-1, TKL-2], (d) orthogroup 639, corresponding to phospho-2-dehydro-3-deoxyheptonate aldolase 1 & 2 [DHS 1 & 2], (e) orthogroup 7791, corresponding to 3-dehydroquinate synthase [DHQS], (f) orthogroup 694, corresponding to 3-dehydroquinate dehydratase/shikimate dehydrogenase [DHQSD], (g) orthogroup 2332, corresponding to shikimate kinase 1 [SK 1], (h) orthogroup 5166, corresponding to 3-phosphoshikimate 1-carboxyvinyltransferase [EPSP], (i) orthogroup 2732, corresponding to chorismate synthase, (j) orthogroup 205, corresponding to phenylalanine ammonia lyase [PAL], (k) orthogroup 851, corresponding to trans-cinnamate 4-monooxygenase [C4H], (l) orthogroup 776, corresponding to hydroxycinnamoyl-CoA quinate hydroxycinnamoyl transferase & hydroxycinnamoyl-CoA shikimate/quinate hydroxycinnamoyl transferase [HQT/HCT], (m) orthogroup 1085, corresponding to *p*-coumaroyl quinate/shikimate 3'-hydroxylase [C3'H], (n) orthogroup 2776, corresponding to caffeoyl-CoA O-methyltransferase [CCoA], (o) orthogroup 2935, corresponding to tapetum-specific methyltransferase 1 [CCoA], (p) orthogroup 396, corresponding to 4-coumarate-CoA ligase 1,2 & 3 [4CL] and (q) orthogroup 11033, corresponding to caffeoyl shikimate esterase (CSE). The figures show rooted trees with plant species belonging to the asterid group that were used as the outgroup. The maximum likelihood (ML) model was used. The clades containing the reference protein and the corresponding orthologs of *Cecropia obtusifolia* are highlighted in green and red boxes, respectively. *C. obtusifolia* unigenes grouped in monophyletic branches might represent unique genes that are sparse due to common redundancies occurring in large-scale transcriptomic projects.

**Figure S6.** Levels of expression in FPKM (fragments by Kilobases of contigs/unigenes per million mapped readings) for the orthologous gene groups identified: (a) orthogroup 7162, corresponding to probable ribose-5-phosphate isomerase 2 [RPI2], (b) orthogroup 8244, corresponding to D-ribulose-5-phosphate 3-epimerase [RPE], (c) orthogroup 1804, corresponding to transketolase 1 & 2 [TKL-1, TKL-2], (d) orthogroup 639, corresponding to phospho-2-dehydro-3-deoxyheptonate aldolase 1 & 2 [DHS & 2], (e) orthogroup 7791, corresponding to 3-dehydroquinate synthase [DHQS], (f) orthogroup 694, corresponding to 3-dehydroquinate dehydratase/shikimate dehydrogenase [DHQSD], (g) orthogroup 2332, corresponding to shikimate kinase 1 [SK 1], (h) orthogroup 694, corresponding to 3-phosphoshikimate 1-carboxyvinyltransferase [EPSP], (i) orthogroup 2732, corresponding to chorismate synthase, (j) orthogroup 205, corresponding to phenylalanine ammonia lyase [PAL], (k) orthogroup 851, corresponding to trans-cinnamate 4-monooxygenase [C4H], (l) orthogroup 776, corresponding to hydroxycinnamoyl-CoA quinate hydroxycinnamoyltransferase & hydroxycinnamoyl-CoA shikimate/quinate hydroxycinnamoyl transferase [HQT/HCT], (m) orthogroup 1085, corresponding to *p-*coumaroyl quinate/shikimate 3'-hydroxylase [C3'H], (n) orthogroup 2776, corresponding to caffeoyl-CoA O-methyltransferase [CCoA], (o) orthogroup 2935, corresponding to tapetum-specific methyltransferase 1 [CCoA], (p) orthogroup 396, corresponding to 4-coumarate-CoA ligase 1,2 & 3 [4CL] and (q) orthogroup 11033, corresponding to caffeoyl shikimate esterase (CSE). The figure shows the levels of gene expression of the corresponding orthologs of *Cecropia obtusifolia* along the kinetics time course established from *C. obtusifolia* cell cultures in suspension. The bars represent the standard deviation calculated for each of the relative expression values of the genes identified as orthologs in *C. obtusifolia* established in the kinetics time course.
